# Author’s reply: MRI in T staging of rectal cancer: How effective is it?

**Published:** 2011

**Authors:** Mubashir Mulla

**Affiliations:** King’s College Hospital, London, United Kingdom. E-mail: drbashir@rediffmail.com

Dear Sir,

We thank the authors Gupta and Gupta[1] for reading our article with keen interest. In response to the question, we have a similar cut off measurement at our institute. We also use “5 mm” as the cut off on MRI for predicting CRM involvement. The CRM is reported as ‘clear’ or ‘not under threat’ if the tumour is beyond 5 mm and ‘involved’ if the tumour is within this distance. We specifically mention this in all our reports with radiological staging.
